# Ultra-High-Resolution Imaging of Rectal Cancer with Photon-Counting Detector Computed Tomography: An Exploratory Pilot Study on the Impact of Slice Thickness and Monoenergetic Reconstruction Level on Image Quality

**DOI:** 10.3390/diagnostics16213697

**Published:** 2026-11-09

**Authors:** Alexander Herold, Andreas Strassl, Daniela Beitzke, Philipp Stelzer, Christian Wassipaul, Theresa Dachs, Bence Nemeth, Florian Lindenlaub, Ahmed Ba-Ssalamah, Johannes Laengle, Michael Bergmann, Michael Weber, Dietmar Tamandl, Michael A. Arnoldner

**Affiliations:** 1 Department of Biomedical Imaging and Image-Guided Therapy, Medical University of Vienna, Waehringer Guertel 18–20, 1090 Vienna, Austria; alexander.herold@meduniwien.ac.at (A.H.); andreas.strassl@meduniwien.ac.at (A.S.); daniela.beitzke@meduniwien.ac.at (D.B.); philipp.stelzer@meduniwien.ac.at (P.S.); theresa.dachs@meduniwien.ac.at (T.D.); bence.nemeth@meduniwien.ac.at (B.N.); florian.lindenlaub@meduniwien.ac.at (F.L.); ahmed.ba-ssalamah@meduniwien.ac.at (A.B.-S.); michael.weber@meduniwien.ac.at (M.W.); michael.arnoldner@meduniwien.ac.at (M.A.A.); 2 Christian Doppler Laboratory for Machine Learning Driven Precision Medicine, Department of Biomedical Imaging and Image-Guided Therapy, Medical University of Vienna, Waehringer Guertel 18–20, 1090 Vienna, Austria; 3 Division of Visceral Surgery, Department of General Surgery, Comprehensive Cancer Center Vienna, Medical University of Vienna, Waehringer Guertel 18-20, 1090 Vienna, Austria; johannes.laengle@meduniwien.ac.at (J.L.); michael.bergmann@meduniwien.ac.at (M.B.)

**Keywords:** ultra-high resolution, photon-counting computed tomography, computed tomography, rectal cancer, image quality, virtual monoenergetic imaging, slice thickness

## Abstract

**Background/Objectives:** To evaluate, in an exploratory pilot study, the influence of slice thickness and monoenergetic (ME) level on quantitative and qualitative image quality of ultra-high-resolution (UHR) photon-counting detector CT (PCD-CT) in rectal cancer (RC). **Methods:** This prospective pilot study enrolled 21 patients with suspected RC undergoing pre-treatment PCD-CT between July 2023 and February 2025. All underwent a QuantumPlus portal venous phase, whose 3 mm/60 keV reconstruction served as the standard reference, plus a separate pelvic UHR acquisition. Sixteen UHR reconstructions were generated (0.6–3 mm; 45–70 keV). Signal-to-noise ratio (SNR), contrast-to-noise ratio (CNR) and noise were analyzed using linear mixed-effects models. Three readers independently rated overall image quality, perceived noise, mesorectal fascia (MRF) sharpness and lesion conspicuity relative to the reference; ratings were analyzed using mixed model ordinal regression. The primary analysis was the within-UHR comparison of reconstruction settings; the comparison with the reference, which differed in contrast phase and acquisition parameters, was descriptive. **Results:** Nineteen patients (mean age 61.7 ± 10.7 years, 73.7% male) were included. CNR showed a significant slice thickness–ME level interaction (*p* < 0.001), ranging from 4.85 ± 2.43 (3 mm/45 keV) to 0.86 ± 1.04 (0.6 mm/70 keV). SNR increased and noise decreased with thicker slices, while both decreased with higher ME levels (all *p* < 0.001). ME level influenced all four qualitative parameters (all *p* < 0.001); slice thickness significantly affected overall quality and perceived noise (*p* < 0.001) but not MRF sharpness or lesion conspicuity (*p* ≥ 0.115). Overall quality was rated favorably in 82.9% at 70 keV versus 45.6% at 45 keV. Lesion conspicuity reached 71.9–86.0% favorable ratings, whereas perceived noise was consistently rated worse than the reference, and UHR SNR remained below the reference in all settings. In a descriptive pooled summary, 3 mm/60 keV (68.0%) and 2 mm/70 keV (66.2%) were rated favorably most often, with overlapping confidence intervals. **Conclusions:** Slice thickness and ME level markedly influence UHR PCD-CT image quality in RC. One candidate working reconstruction for future validation is 3 mm at 60 keV, with 2 mm at 70 keV as a plausible alternative. Because the reference differed in acquisition and contrast phase and no diagnostic endpoint was assessed, these results do not establish a diagnostic benefit of UHR PCD-CT.

## 1. Introduction

Colorectal cancer ranks third in incidence and second in cancer-related mortality worldwide [1]. Rectal cancer (RC) accounts for about one-third of cases, with five-year survival falling from over 90% in stage I to around 15% in stage IV disease [2,3].

Current guidelines from the European Society of Gastrointestinal and Abdominal Radiology (ESGAR) and the National Comprehensive Cancer Network (NCCN) recommend pelvic magnetic resonance imaging (MRI) for local staging and computed tomography (CT) of the chest and abdomen for assessment of distant metastases [4,5]. Despite its broad availability, conventional energy-integrating detector CT (EID-CT) has not gained clinical relevance for primary tumor evaluation in RC, mainly because integrating the total energy of incoming photons yields lower contrast resolution than MRI, together with electronic noise and limited spatial resolution [6,7].

Recent technological advances have introduced photon-counting detector CT (PCD-CT), which directly converts individual X-ray photons into electrical signals and enables energy discrimination [6,7,8]. Compared to EID-CT, PCD-CT offers improved spatial resolution (detector pixel size 0.2 × 0.2 mm at isocenter in ultra-high-resolution (UHR) mode) and higher contrast-to-noise and signal-to-noise ratios [8]. Early clinical experience in abdominal oncologic imaging has demonstrated potential for improved detection and characterization of upper abdominal neoplasms [9,10,11,12] and for response assessment in pancreatic ductal adenocarcinoma [13] and RC [14]. For RC, where fine anatomical detail is essential for staging, UHR mode may address the limitations of conventional CT.

In RC, Zhu et al. recently optimized monoenergetic (ME) level and quantum iterative reconstruction strength in standard acquisition mode and reported T-staging performance comparable to MRI [15]. To date, however, very few studies have applied the UHR mode in abdominal oncologic imaging, and to our knowledge, no study has systematically evaluated quantitative and qualitative parameters of UHR photon-counting CT in RC. Before UHR PCD-CT can be tested against MRI or histopathology, the reconstruction settings to be used in such a diagnostic study need to be defined, because the sharper kernel and larger matrix of UHR mode change the noise behavior of virtual monoenergetic images and settings optimized in standard mode may not transfer.

The objective of this exploratory, prospective pilot study was therefore to characterize the quantitative and qualitative image quality of UHR PCD-CT in patients with suspected RC as a function of slice thickness and ME reconstruction level, and to identify candidate reconstruction settings for subsequent diagnostic validation. The study did not assess diagnostic performance.

## 2. Materials and Methods

### 2.1. Study Design and Patient Population

This prospective, single-center observational pilot study was conducted between July 2023 and February 2025. The study was approved by the Ethics Committee of the Medical University of Vienna (EK No. 1378/2023) and conducted in accordance with the Declaration of Helsinki. Written informed consent was obtained from all participants. Patients aged ≥18 years were enrolled if they had clinically suspected (endoscopy and/or prior MRI) or histologically confirmed primary RC and were scheduled for routine staging CT of the abdomen and pelvis. Exclusion criteria were glomerular filtration rate < 30 mL/min/1.73 m^2^, severe reaction to iodinated contrast, significant metallic artifacts in the field of view, withdrawal of consent, and pregnancy. Because enrollment was based on clinical suspicion, the final histological diagnosis was established after imaging; patients whose final diagnosis was not rectal adenocarcinoma were retained in the main analysis and addressed in a sensitivity analysis (Section 2.4). No formal sample size calculation was performed and no primary endpoint was prespecified; enrollment was limited to eligible patients presenting during the study period, in keeping with the exploratory pilot design, and all analyses are to be regarded as exploratory. The study is reported in accordance with the STROBE statement.

### 2.2. Image Acquisition and Reference Reconstruction

All examinations were performed on a first-generation photon-counting detector scanner (NAEOTOM Alpha, Siemens Healthineers, Erlangen, Germany). Patients received a staging CT of the abdomen and pelvis in QuantumPlus mode, comprising a portal venous phase (absolute delay 77 s; 144 × 0.4 mm collimation; CARE keV image quality level 170) and additional regions as clinically requested (e.g., chest, arterial phase of the upper abdomen), after weight-adjusted iodinated contrast (Iomeron 400, Bracco Imaging, Milan, Italy; 1 mL/kg body weight). In addition to, not instead of, this standard examination, a pelvic scan covering only the rectum was performed in UHR mode (HighResUltra; 120 × 0.2 mm collimation; CARE keV image quality level 250; 768 matrix; Br48 kernel) with an absolute delay of 40 s after the start of contrast injection, i.e., before the portal venous phase. All acquisition and reconstruction parameters are listed in Table 1.

The standard reference against which the UHR reconstructions were compared was the 3 mm, 60 keV reconstruction of the portal venous QuantumPlus phase, which is the series used for rectal assessment in our routine staging reports. This reference therefore differed from the UHR series not only in acquisition mode but also in contrast phase, collimation, reconstruction kernel, matrix size and tube output (Table 1). The comparison between UHR reconstructions and the reference is consequently a comparison between two clinical series rather than an isolated test of the UHR mode, and it was treated as descriptive throughout. The primary analysis of this study is the comparison among the sixteen UHR reconstructions, which were all derived from the same raw data, so that their quantitative comparison is not affected by these acquisition differences.

### 2.3. Image Analysis

#### 2.3.1. Quantitative Measurements

Four ME reconstruction levels (45, 50, 60 and 70 kiloelectron volt (keV)) were each reconstructed at four slice thicknesses (0.6, 1, 2 and 3 mm), giving sixteen combinations per patient. Region-of-interest (ROI) measurements were performed by one reader (A.H.) on a dedicated workstation. A circular ROI of approximately 50 mm^2^ was placed in the largest solid portion of the tumor on the axial slice with the greatest tumor diameter, avoiding necrosis, intraluminal gas and vessels; a second ROI of the same size was placed in the right psoas muscle at the same level as reference tissue. Because all sixteen UHR reconstructions were derived from the same acquisition and share identical geometry, the ROIs were copied to identical positions and sizes across reconstructions, so that differences between reconstructions reflect reconstruction settings rather than ROI placement; in the reference reconstruction the ROIs were placed at the corresponding anatomical positions. For each ROI, mean attenuation (in Hounsfield units, HU) and standard deviation of tumor and muscle were recorded. Standard deviation in muscle was defined as noise. Signal-to-noise ratio (SNR) was calculated by mean tumor attenuation divided by standard deviation of muscle, and contrast-to-noise ratio (CNR) by mean tumor minus mean muscle divided by standard deviation of muscle. Intra- or inter-observer reproducibility of the ROI measurements was not assessed.

#### 2.3.2. Qualitative Reader Assessment

Qualitative image analysis was performed by three senior radiology residents with 5, 5 and 4 years of experience in cross-sectional oncologic imaging. Residents were chosen as readers by design: at our institution, residents produce the primary report of staging CT examinations, so they are the readers who will use a reconstruction protocol in daily practice; protocol optimization has been shown to benefit less experienced readers most [15]; the rating task was perceptual (noise, sharpness, conspicuity) rather than diagnostic; and residents are less habituated to the appearance of the established reference reconstruction, which reduces the risk of a familiarity bias in favor of the reference. The readers were not informed of the reconstruction parameters, but had information about the diagnosis of RC and the standard reference (QuantumPlus mode, 3 mm, 60 keV). All sixteen reconstructions per patient were presented in axial orientation in randomized order using pseudonymized identifiers on a dedicated imaging workstation. The assessment parameters included overall image quality, perceived noise levels, sharpness of the mesorectal fascia (MRF) and lesion conspicuity. All parameters were evaluated using a standardized five-point Likert scale ranging from −2 (markedly inferior to the reference) through −1 (moderately inferior), 0 (equivalent), +1 (moderately superior) to +2 (markedly superior to the reference). Detailed scoring definitions are given in Table S1. Locoregional lymph nodes, extramural vascular invasion and T category were deliberately not assessed: the study addressed image quality of the primary tumor and the MRF, and a per-node or per-patient assessment of these features would have required a histopathological or MRI reference standard that was outside the scope of this image-quality study.

### 2.4. Statistical Analysis

Statistical analysis was performed using R version 4.5.1 (R Foundation for Statistical Computing, Vienna, Austria) and SPSS version 28.0 (IBM Corporation, Armonk, NY, USA); cluster-bootstrap confidence intervals, per-patient confidence intervals and the power estimate were computed in Python version 3.13 (Python Software Foundation, Wilmington, DE, USA) with the statsmodels (version 0.15) and SciPy (version 1.18) libraries. Descriptive data are presented as mean ± standard deviation, median (interquartile range) or absolute number (percentage), as appropriate.

Linear mixed-effects models with patient as a random intercept were used to evaluate the effects of slice thickness (0.6, 1, 2, 3 mm) and ME level (45, 50, 60, 70 keV) on quantitative image quality parameters, including their interaction. Fixed effects were tested with Type III F tests and effect sizes are reported as partial eta squared (η^2^p). To convey the magnitude and precision of the effects, estimated marginal means of SNR, CNR and noise for each slice thickness (averaged over ME levels) and each ME level (averaged over slice thicknesses) and their differences from 3 mm and from 60 keV, respectively, as well as two selected contrasts (1 mm versus 0.6 mm, and 3 mm/60 keV versus 0.6 mm/70 keV, the thinnest slice at the highest ME level), are reported with 95% confidence intervals (CI) calculated from the 19 per-patient values (t distribution, 18 degrees of freedom), so that their precision reflects the number of independent patients rather than the number of reconstructions. The QuantumPlus reference reconstruction was reported descriptively and was not entered into the inferential models.

For qualitative assessment, cumulative link mixed models with a logit link were used. Each rating was modeled as an ordered five-point outcome, with slice thickness and ME level as categorical fixed effects including their interaction, and random intercepts for both patient and reader to account for repeated ratings of the same patient and repeated ratings by the same reader. Main and interaction effects were tested by likelihood-ratio tests against the corresponding nested model. The proportional-odds assumption was checked by nominal-effects tests. Because it was not met for every parameter, effects are reported as likelihood-ratio tests rather than as single odds ratios, and these tests are interpreted only as indicating whether a factor changed the distribution of ratings, not as a uniform shift along the rating scale; the direction and size of the effects are described by the proportions of favorable and markedly superior ratings (Table S2), which do not rely on this assumption. Two sensitivity analyses that do not require this assumption, mixed logistic regression of favorable (+1 or +2) versus other ratings and Wilcoxon signed-rank tests of per-patient mean ratings, yielded the same conclusions. The proportion of ratings in which each combination was rated superior to the standard reference (favorable, +1 or +2) was calculated across the three readers and is reported with 95% CIs obtained by a cluster bootstrap that resampled patients with all their ratings (2000 resamples, percentile method), thereby respecting the clustering of ratings within patients. As a descriptive summary only, the four qualitative parameters were additionally pooled with equal weighting (“all parameters combined”); this pooled measure does not imply that the parameters are of equal clinical importance and is not a validated composite endpoint.

Because no primary endpoint was prespecified, candidate reconstruction settings for future validation were identified post hoc and transparently as the combinations with the highest pooled proportions of favorable ratings, with the four individual parameters and the quantitative measures used to describe their trade-offs; given the descriptive nature of the pooled measure, this ranking was not interpreted as identifying a uniquely optimal setting. No multiplicity adjustment was applied across the four qualitative and three quantitative endpoints, and all *p* values should be interpreted as exploratory. As a sensitivity analysis addressing the heterogeneity of the cohort, the quantitative mixed models and the favorable-rating proportions were recalculated after exclusion of the two patients whose final diagnosis was not rectal adenocarcinoma.

Inter-reader agreement was assessed using Gwet’s agreement coefficient version 2 (AC2) with ordinal weights [16]. Altman’s criteria, originally proposed for kappa, were applied to characterize agreement (0.8–1.0 very good, 0.6–0.8 good, 0.4–0.6 moderate, 0.2–0.4 fair, below 0.2 poor), with labels assigned using the lower 95% confidence interval estimate.

To illustrate the limited precision of the study, a simplified calculation was performed: with 19 patients, a paired comparison between two reconstruction settings would have 80% power at a two-sided α of 0.05 to detect a standardized within-patient difference of d ≥ 0.68. This calculation does not apply directly to the mixed-effects analyses and does not establish that the study was adequately powered for them; the study was underpowered for small differences between neighboring settings and for any diagnostic endpoint. Statistical significance was set at *p* < 0.05 for all analyses.

## 3. Results

### 3.1. Patient Demographics, Tumor Characteristics and Radiation Metrics

Of 21 enrolled patients, two were excluded because of significant metallic artifacts from hip replacements in the field of view, leaving 19 patients (mean age 61.7 ± 10.7 years, 14 (73.7%) male) for analysis (Figure S1). Adenocarcinoma was the predominant histology (17 patients, 89.5%); the final diagnosis was high-grade intraepithelial neoplasia in one patient and anal squamous cell carcinoma in one patient, both enrolled on clinical suspicion of RC. Most patients with rectal adenocarcinoma presented with locally advanced disease, with 11 (64.7%) classified as American Joint Committee on Cancer stage IIIB and 14 (82.4%) demonstrating positive nodal involvement. Tumors were equally distributed between the lower and middle thirds (42.1% each), with three patients (15.8%) having upper third tumors, and the MRF was involved (tumor-to-fascia distance ≤1 mm) in 5 of 17 adenocarcinomas (29.4%). Of the 17 adenocarcinomas, four (23.5%) were polypoid, 10 (58.8%) semi-annular and three (17.6%) annular on the staging MRI, and none was a mucinous adenocarcinoma. Complete characteristics are presented in Table 2.

The mean volume computed tomography dose index (CTDIvol) of the UHR pelvic acquisition was 18.3 ± 2.9 mGy, with a dose length product (DLP) of 343.0 ± 144.9 mGy·cm and a size-specific dose estimate of 22.7 ± 2.9 mGy, approximately double the CTDIvol of the QuantumPlus portal venous (8.4 ± 2.5 mGy) and arterial (8.7 ± 3.0 mGy) phases. The mean total DLP across all series was 1134.6 ± 364.6 mGy·cm (Table 3); the additional UHR acquisition thus accounted for 30% of the total DLP of the examination, corresponding to an additional effective dose of approximately 5.1 mSv (range 1.8–11.7 mSv) when a conversion coefficient of 0.015 mSv·mGy^−1^·cm^−1^ for the adult abdomen and pelvis is applied [17].

### 3.2. Quantitative Parameters

#### 3.2.1. Effects of Slice Thickness and ME Level Within UHR Mode

**Figure 1:**
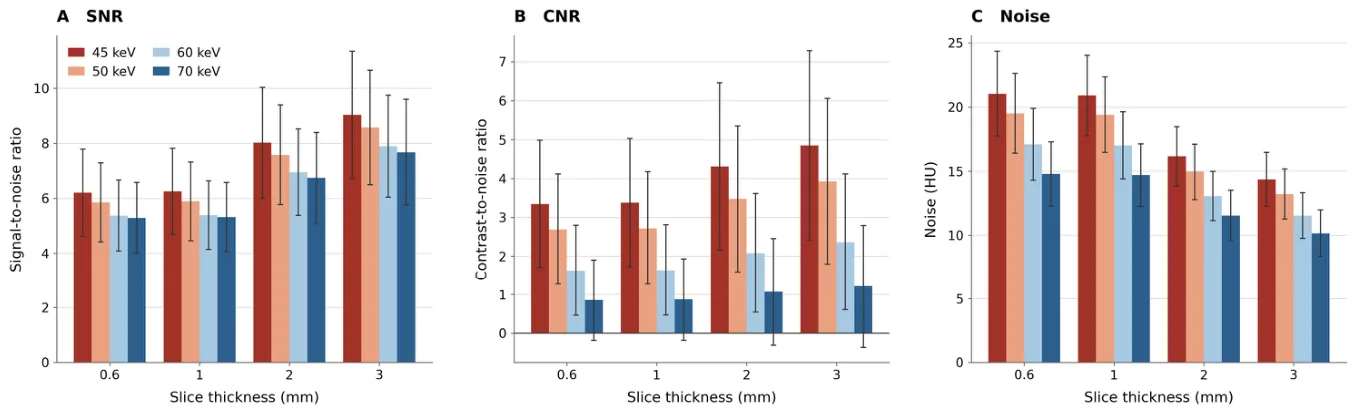
Quantitative parameters by slice thickness and monoenergetic level. Bars show mean values; error bars show one standard deviation. (**A**) Signal-to-noise ratio. (**B**) Contrast-to-noise ratio. (**C**) Noise. CNR, contrast-to-noise ratio; keV, kiloelectron volt; SNR, signal-to-noise ratio.

**Figure 2:**
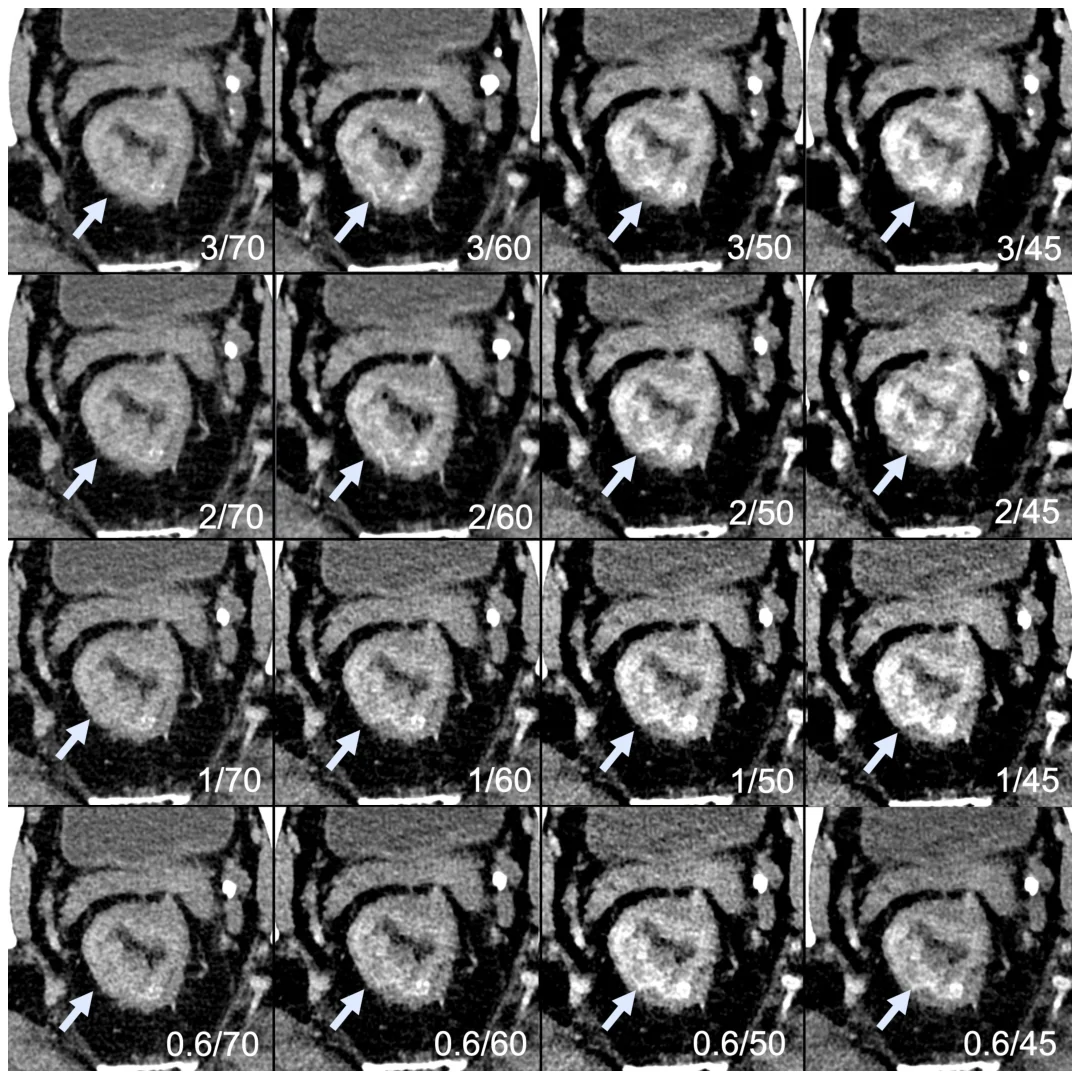
Ultra-high-resolution photon-counting CT reconstructions in a patient with rectal adenocarcinoma (arrows). The 4 × 4 grid shows all sixteen combinations of slice thickness (3.0–0.6 mm, top to bottom) and monoenergetic level (70–45 keV, left to right). Lower monoenergetic levels increase contrast between tumor and surrounding tissue at the expense of image noise, whereas higher levels reduce noise with lower tumor contrast. Thicker slices reduce noise, while thinner slices resolve finer detail of the rectal wall and mesorectal fascia with correspondingly higher noise. keV, kiloelectron volt.

SNR, CNR and noise varied significantly across slice thicknesses and ME levels (Table 4, Table 5 and Table 6, Figure 1); representative images of all sixteen reconstructions in one patient are shown in Figure 2.

CNR between tumor and muscle tissue was influenced by both slice thickness (*p* < 0.001, η^2^p 0.33) and ME level (*p* < 0.001, η^2^p 0.52). Higher CNR values were achieved with lower ME levels and greater slice thicknesses. Slice thickness and ME level also interacted (*p* < 0.001, η^2^p 0.15). CNR values ranged from 4.85 ± 2.43 at 3.0 mm/45 keV to 0.86 ± 1.04 at 0.6 mm/70 keV (Table 4). At 70 keV, tumor attenuation fell below that of psoas muscle in four patients, resulting in negative CNR values, whereas CNR was positive in all patients at 45 and 50 keV.

SNR increased with greater slice thickness (*p* < 0.001, η^2^p 0.67) and decreased with higher ME levels (*p* < 0.001, η^2^p 0.16). These two factors did not interact with each other (*p* = 0.455). SNR values ranged from 5.29 ± 1.29 at 0.6 mm/70 keV to 9.03 ± 2.31 at 3.0 mm/45 keV.

Noise measurements showed that both increasing slice thickness (*p* < 0.001, η^2^p 0.79) and higher ME levels (*p* < 0.001, η^2^p 0.72) resulted in decreased noise values, with a significant interaction between both factors (*p* < 0.001, η^2^p 0.18). Mean noise values ranged from 21.03 ± 3.31 at 0.6 mm/45 keV to 10.15 ± 1.81 at 3.0 mm/70 keV.

The magnitude of these effects is summarized in Table 6. Averaged over ME levels, 3 mm slices compared with 0.6 mm slices increased SNR by 2.62 (95% CI 2.25–2.98) and CNR by 0.95 (95% CI 0.63–1.28) and reduced noise by 5.79 (95% CI 5.13–6.44) HU, whereas 1 mm and 0.6 mm slices did not differ meaningfully (SNR difference 0.03 (95% CI −0.06 to 0.13); CNR 0.02 (95% CI −0.03 to 0.07); noise −0.10 (95% CI −0.42 to 0.22) HU). Averaged over slice thicknesses, 45 keV compared with 60 keV increased CNR by 2.04 (95% CI 1.70–2.38) at the cost of 3.44 (95% CI 3.05–3.83) HU higher noise, whereas 70 keV reduced noise by 1.88 (95% CI 1.57–2.19) HU but lowered CNR by 0.92 (95% CI 0.80–1.05). After exclusion of the two patients without rectal adenocarcinoma (*n* = 17), all main effects remained significant (all *p* < 0.001) with η^2^p values equal to or up to 0.05 larger than those of the full cohort, and the interaction pattern was unchanged (Table S4).

#### 3.2.2. Descriptive Comparison with the Standard Reference Reconstruction

Across all UHR settings, SNR remained lower and noise higher than in the QuantumPlus reference reconstruction (SNR 11.12 ± 2.82; noise 8.30 ± 1.34), whereas CNR exceeded the reference value of 2.73 ± 2.12 at 45 keV at every slice thickness and at 50 keV with 2 and 3 mm slices; at 50 keV with 0.6 and 1 mm slices it was similar to the reference (2.69 and 2.72). As noted in Section 2.2, these differences reflect the combined effect of acquisition mode, contrast phase and reconstruction parameters and cannot be attributed to UHR mode alone.

### 3.3. Qualitative Parameters

**Figure 3:**
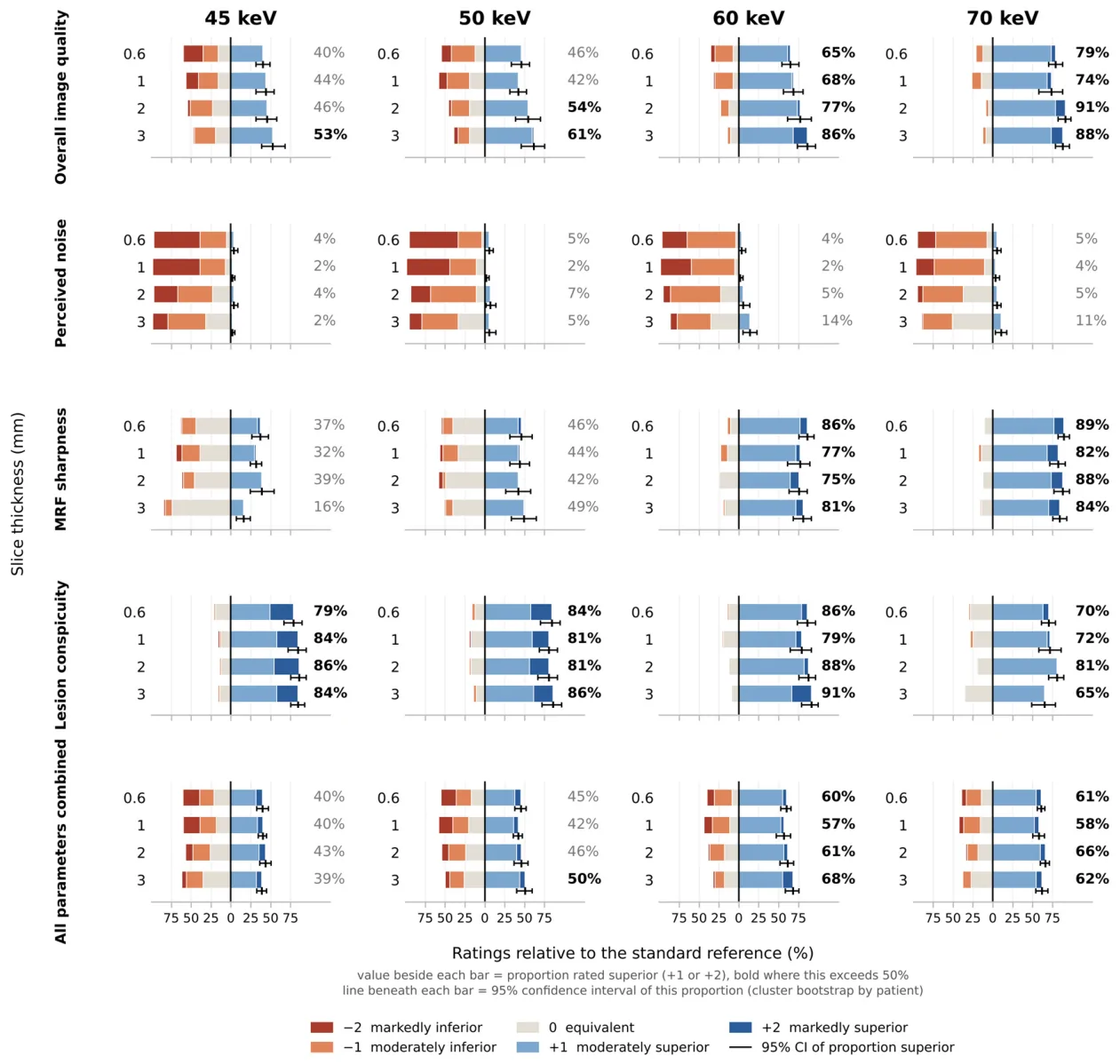
Distribution of qualitative ratings of ultra-high-resolution reconstructions relative to the standard reference (QuantumPlus, 3 mm, 60 keV). Diverging bars are centered on the equivalence rating; ratings worse than the reference extend to the left and better ratings to the right. The value beside each bar gives the proportion rated superior (+1 or +2), shown in bold where this exceeds 50%; the line beneath each bar shows the 95% confidence interval of this proportion obtained by a cluster bootstrap over patients. “All parameters combined” pools the four assessment parameters with equal weighting as a descriptive summary; it is not a validated composite endpoint. keV, kiloelectron volt; MRF, mesorectal fascia.

Performance relative to the standard reference varied across ME levels and slice thicknesses for all four parameters; the full distribution of ratings is shown in Figure 3 and Table S2, and the proportion of favorable ratings with 95% CIs for every reconstruction and for each ME level pooled over slice thicknesses is given in Table S5.

Overall image quality was influenced by both ME level and slice thickness (both *p* < 0.001) without a significant interaction (*p* = 0.344; Table 7). Across all slice thicknesses, 82.9% (95% CI 75.4–89.0%) of ratings were favorable (+1, +2) at 70 keV versus 45.6% (95% CI 36.4–55.3%) at 45 keV.

Perceived noise was influenced by both ME level and slice thickness (both *p* < 0.001) without interaction (*p* = 0.978), and was rated worse than the standard reference at all ME levels (favorable in 2.6% to 6.1%; upper 95% confidence limits ≤ 10.1%).

MRF sharpness was influenced by ME level (*p* < 0.001) but not significantly by slice thickness (*p* = 0.115), without interaction (*p* = 0.948), and varied markedly between ME levels, from 30.7% (95% CI 22.4–39.0%) favorable at 45 keV to 79.8% (95% CI 69.7–87.3%) at 60 keV and 86.0% (95% CI 80.3–91.2%) at 70 keV.

Lesion conspicuity was influenced by ME level (*p* < 0.001) but not significantly by slice thickness (*p* = 0.361), without interaction (*p* = 0.115). Favorable ratings remained high at every ME level (71.9% to 86.0%), but on the full ordinal scale conspicuity declined monotonically with increasing ME level, with markedly superior (+2) ratings falling from 28.5% at 45 keV to 2.6% at 70 keV. This pattern, high proportions of favorable ratings at every ME level combined with a steep decline in markedly superior ratings, is not a uniform shift along the rating scale and illustrates why the effects are described by proportions rather than by single odds ratios (Section 2.4).

In the descriptive summary pooling all four qualitative parameters, 60 and 70 keV achieved 61.5% (95% CI 53.8–67.7%) and 61.7% (95% CI 56.5–66.0%) favorable ratings, respectively, compared to 40.6% (95% CI 35.0–45.8%) at 45 keV and 45.9% (95% CI 38.7–52.3%) at 50 keV. Among the sixteen reconstructions, 3 mm at 60 keV had the highest pooled proportion of favorable ratings (68.0%, 95% CI 58.8–75.9%), followed closely by 2 mm at 70 keV (66.2%, 95% CI 60.1–71.5%); the confidence intervals of the leading combinations overlapped widely (Table S5), so this ranking does not identify a uniquely optimal setting. After exclusion of the two patients without rectal adenocarcinoma, the two leading combinations were unchanged: 3 mm at 60 keV remained the combination with the highest pooled proportion of favorable ratings (69.6%), followed by 2 mm at 70 keV (66.7%; Table S4).

### 3.4. Inter-Reader Agreement

Inter-reader agreement ranged from moderate to very good using Altman’s benchmarking criteria applied to the lower limits of the 95% CIs: perceived noise moderate (AC2 0.64, 95% CI: 0.59–0.69), overall image quality good (AC2 0.72, 95% CI: 0.68–0.75), MRF sharpness very good (AC2 0.83, 95% CI: 0.80–0.85) and lesion conspicuity very good (AC2 0.88, 95% CI: 0.87–0.90). Details are given in Table S3.

## 4. Discussion

In this exploratory prospective pilot study, we evaluated four slice thicknesses (0.6–3 mm) and four ME levels (45–70 keV) in UHR mode in patients with RC, and found that reconstruction settings substantially affected both objective and subjective image quality. Within UHR mode, the effects of slice thickness and ME level were large. Relative to our standard reference reconstruction (3 mm, 60 keV in QuantumPlus mode), most UHR reconstructions received favorable ratings for overall quality and lesion conspicuity, and those at 60 and 70 keV also for MRF sharpness, whereas perceived noise was consistently rated worse and measured SNR was lower in all settings; because the reference differed in contrast phase and acquisition parameters, this comparison describes two clinical series rather than the isolated effect of UHR mode.

Several studies have assessed reconstruction parameters in abdominal oncologic photon-counting CT. In pancreatic ductal adenocarcinoma, virtual ME reconstructions at low keV improved tumor conspicuity, which was highest at 40 keV, and yielded a higher CNR than conventional CT [10]. In contrast, in a phantom study of liver lesion detection with photon-counting CT, detectability was highest at 65 and 70 keV [18]. Szelenyi et al. recently evaluated hepatocellular carcinoma in QuantumPlus mode and showed that reconstruction kernel and slice thickness exerted a significant influence on image quality [12]. Beyond these standard acquisitions, in the vascular setting, Zhang et al. demonstrated that UHR reconstructions improve arterial sharpness and delineation [19], and Ayx et al. confirmed the feasibility of UHR abdominal CT angiography [20], emphasizing both the potential for higher spatial resolution and the trade-off of increased image noise. For RC, reconstruction settings have so far been optimized only in standard acquisition mode, by Zhu et al., whose findings we return to below [15]. Collectively, these studies underline the relevance of UHR mode in abdominal imaging, but data in oncologic applications remain scarce. The incremental contribution of the present study is therefore not the direction of the effects, which follow from first principles, but their magnitude in UHR mode in RC and the observation that readers favored 60–70 keV for overall image quality in UHR mode, whereas 40 keV, which was not tested here, was identified for staging with standard-mode PCD-CT of RC [15].

In this study we applied UHR mode in RC imaging at a fixed reconstruction kernel and systematically assessed the influence of slice thickness and ME level. Quantitative analysis revealed a significant interaction between slice thickness and ME level for CNR and noise, indicating a complex moderation in which the effect of one parameter depended on the other, whereas SNR demonstrated predictable main effects only, increasing with slice thickness and decreasing with higher ME levels. The effect estimates in Table 6 show that these effects were not only statistically significant but substantial: moving from 0.6 mm to 3 mm reduced the noise by about one-third, and moving from 60 to 45 keV doubled the CNR at the cost of a quarter more noise. Compared with the standard reference, the UHR reconstructions showed consistently lower SNR and higher noise, as expected from the sharper kernel and higher matrix, whereas tumor-to-muscle CNR exceeded the reference at 45 keV at every slice thickness and at 50 keV with 2 and 3 mm slices; the earlier contrast phase of the UHR acquisition (40 s versus 77 s) may have contributed to the latter.

**Figure 4:**
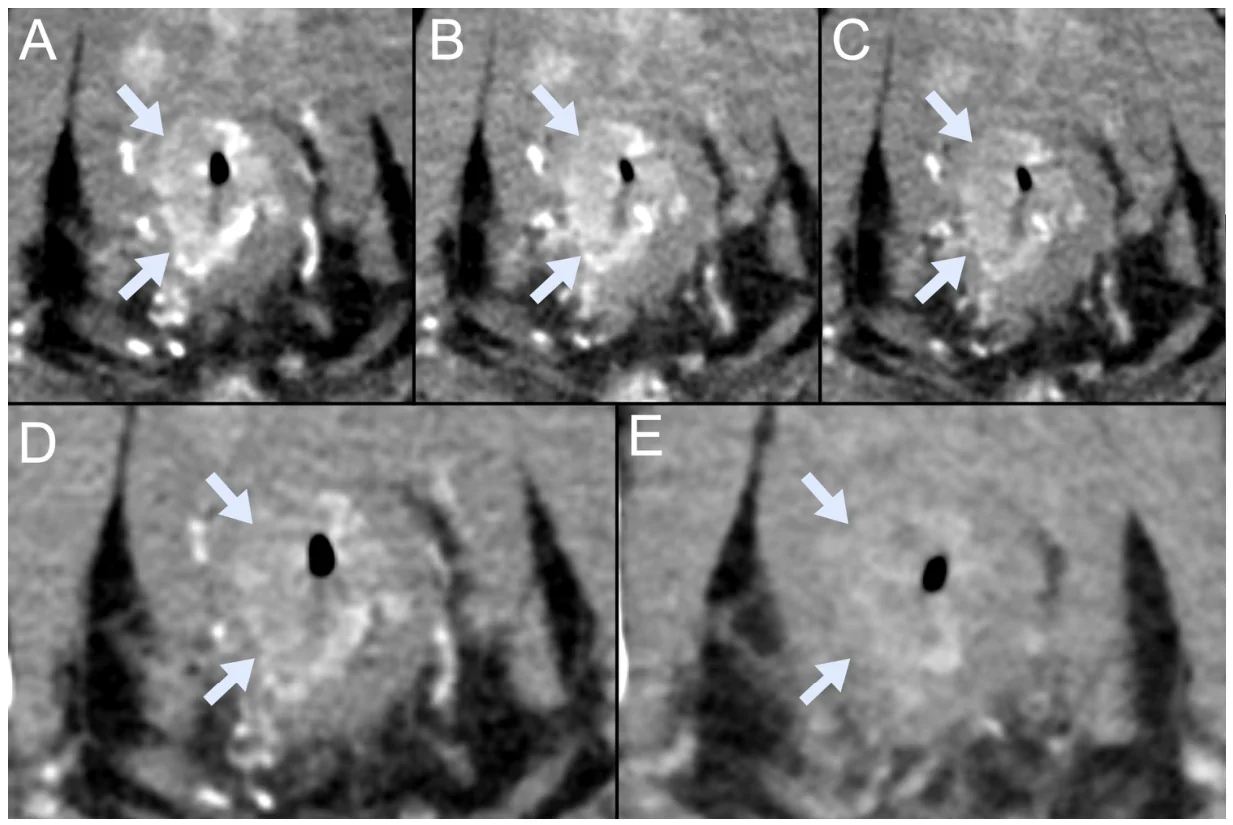
Representative ultra-high-resolution photon-counting CT images in a patient with adenocarcinoma of the lower rectal third (arrows). (**A**–**D**) Ultra-high-resolution reconstructions at 3 mm slice thickness and 45, 50, 60 and 70 keV. (**E**) Standard reference reconstruction (QuantumPlus, 3 mm, 60 keV). Tumor-to-muscle contrast is highest in (**A**,**B**) and decreases toward (**D**), while perceived noise decreases in the same direction; in this example, the mesorectal fascia appears more sharply delineated in (**C**,**D**) than in the reference image (**E**), which was acquired in a different contrast phase. keV, kiloelectron volt.

In qualitative assessment, most UHR reconstructions at 60 and 70 keV were rated equal to or better than the standard reference for overall image quality, MRF sharpness and lesion conspicuity, although perceived noise was consistently rated worse across reconstruction combinations. Readers thus preferred many UHR reconstructions despite their lower SNR and higher noise, indicating that noise-based metrics alone do not capture perceived image quality, a dissociation also reported for sharper kernels in hepatic PCD-CT [12]. Because every UHR reconstruction was rated against the same anchor, the differences between UHR settings, which form the primary analysis of this study, depend much less on the acquisition differences between UHR series and reference than does the absolute level of the ratings, which should not be read as evidence of superiority of UHR mode as such. In the pooled descriptive summary, 60 and 70 keV received a majority of favorable ratings when averaged over slice thicknesses (61.5% and 61.7%, respectively), whereas 45 and 50 keV did not (40.6% and 45.9%, respectively). A representative example is shown in Figure 4. Notably, overall image quality and MRF sharpness improved at higher ME levels, whereas the proportion of markedly superior lesion conspicuity ratings was highest at the lower ME levels.

The quantitative and qualitative endpoints point in different directions: lower ME levels maximize tumor-to-muscle CNR and the proportion of markedly superior lesion conspicuity ratings, higher ME levels minimize noise and improve overall quality and MRF sharpness, and thicker slices reduce noise at the expense of through-plane resolution. These endpoints are not interchangeable and their relative clinical weight is unknown. We therefore did not attempt a weighted optimization but applied a simple, transparent and post hoc criterion, the highest pooled proportion of favorable ratings, which identified 3 mm at 60 keV (68.0%, 95% CI 58.8–75.9%), followed closely by 2 mm at 70 keV (66.2%, 95% CI 60.1–71.5%). The two settings reached similar pooled proportions by different routes: 3 mm at 60 keV had the highest proportion of favorable lesion conspicuity ratings (91.2%) and a higher tumor-to-muscle CNR, whereas 2 mm at 70 keV, with thinner slices and the same mean measured noise (Table 4), was rated favorably more often for overall image quality (91.2% versus 86.0%) and MRF sharpness (87.7% versus 80.7%); for both, perceived noise was rated below the reference in about half or more of the ratings (50.9% and 57.9%). Because the pooled measure has no established clinical weighting and the confidence intervals of these and several neighboring settings, including 3 mm at 70 keV, overlapped widely, the ranking does not establish a uniquely optimal reconstruction, and the highest pooled proportion is not evidence of clinical superiority or of future diagnostic performance. We therefore regard 3 mm at 60 keV as one candidate working reconstruction for future validation and 2 mm at 70 keV as a plausible alternative; both should be carried forward into a diagnostic study, and neither is a validated recommendation for RC staging. Where fine anatomical detail is required, a thin-slice series could be evaluated in addition, since MRF sharpness did not differ significantly between slice thicknesses and was rated favorably most often at 0.6 mm and 70 keV; because 0.6 mm and 1 mm did not differ meaningfully in any quantitative measure (Table 6), a 1 mm series would limit the data volume.

Our candidate settings differ from those of Zhu et al., who optimized ME level and iterative reconstruction strength for RC T staging in standard acquisition mode, identified 40 keV with maximal iterative reconstruction as optimal, and demonstrated T-staging performance comparable to MRI [15]. The difference is informative rather than contradictory: markedly superior lesion conspicuity ratings in our study were likewise most frequent at the lower ME levels, but their soft kernel and maximal iterative reconstruction suppress the noise penalty of low ME levels, whereas our sharper, higher-matrix UHR reconstruction amplifies it. The optimal ME level is therefore likely a property of the whole reconstruction chain, and settings validated in standard mode may not transfer directly to UHR imaging; 40 keV was not tested in the present study.

The additional UHR acquisition carried a radiation cost. Its CTDIvol was about twice that of the portal venous phase and it added a mean DLP of 343 mGy·cm, 30% of the total examination DLP, corresponding to an additional effective dose of about 5 mSv [17]. This study did not demonstrate a diagnostic gain that would justify this exposure, and no such claim is made. Whether the clinical benefit of UHR PCD-CT outweighs the additional dose can only be answered by a diagnostic study that links image quality to staging accuracy, and the dose should be an explicit endpoint of that study. Two design features would reduce the exposure substantially: acquiring the UHR series in place of, rather than in addition to, the pelvic portion of the standard scan, and lowering the image quality level of the UHR acquisition, since the dose penalty of UHR mode stems largely from the elevated image quality level needed to compensate for the smaller detector elements [6]. The present data narrow down the reconstruction settings to be evaluated in such a dose-neutral design.

The clinical implications of our findings are accordingly limited. We did not assess T category, nodal status, extramural vascular invasion or MRF involvement against histopathology or MRI, and improved visualization of the tumor and the MRF does not establish that these features can be assessed reliably. MRI remains the reference standard for local staging of RC. Whether UHR PCD-CT could contribute to staging in patients in whom MRI is contraindicated or not feasible remains untested and would require a dedicated diagnostic study.

### Limitations

This study has limitations. First, this was a single-center pilot study with a relatively small sample size of 19 patients and was underpowered: the detectable-effect estimate given in Section 2.4 (d ≥ 0.68 for a simplified paired comparison) illustrates its limited precision and does not imply adequate power for the mixed-effects analyses. Small differences between neighboring reconstruction settings, as reflected by the overlapping confidence intervals of the leading combinations, could not be resolved, and no diagnostic endpoint was included. In the future, larger study groups must be examined to validate the findings reported here. Sample estimates of future efforts need to utilize 85 subjects at a power of 0.80 and alpha = 0.05 for the primary endpoint of such a study, the paired comparison of per-patient T-category accuracy (T1–2 versus T3–4) between a prespecified candidate UHR reconstruction and the standard reference reconstruction against histopathology in patients scheduled for primary surgery, assuming correct classification in 65% and 80% of patients, values that approximate the areas under the curve for T3–T4 staging reported by Zhu et al. for standard (0.64) and optimized (0.80–0.82) PCD-CT reconstructions read by less experienced radiologists [15], and 25% discordant pairs (McNemar test); allowing for 15% of patients without a histopathological reference, approximately 100 patients should be enrolled.

Second, the reference reconstruction was acquired in a different contrast phase and with different collimation, kernel, matrix and tube output than the UHR series. This is intrinsic to the design, which compared the added UHR series with the series actually used for rectal assessment in our clinical routine, and it means that the ratings relative to the reference, as well as the quantitative comparison with the reference, cannot be attributed to the UHR mode alone. The within-UHR comparisons form the primary analysis; their quantitative part is not affected by this confounding, and because the ratings of the UHR settings share the same anchor, their ranking is much less affected than their absolute level, although an influence of the anchor cannot be excluded.

Third, the readers were senior radiology residents rather than subspecialists, by design (Section 2.3.2); their agreement was good to very good for three of the four parameters. Nevertheless, specialist readers may weight the trade-off between noise and sharpness differently, so the preferences reported here should not be generalized to subspecialist staging without confirmation, and the confirmatory study will include subspecialist readers. Fourth, readers could not be blinded to acquisition mode, as UHR reconstructions are visually distinguishable, so an expectation bias favoring UHR cannot be excluded.

Fifth, the study addressed image quality rather than diagnostic performance. No direct comparison with MRI or histopathology was performed, lymph nodes and extramural vascular invasion were not assessed, and MRI remains the reference standard for local staging. The candidate reconstruction settings were derived from a post hoc criterion applied to competing endpoints and are not validated recommendations. Sixth, the cohort was heterogeneous: it included one patient with high-grade intraepithelial neoplasia and one with anal squamous cell carcinoma, both enrolled on clinical suspicion of RC; excluding these patients did not change the quantitative effects or the leading reconstructions in the pooled qualitative assessment (Table S4). Tumor morphology, which may influence lesion conspicuity, could not be analyzed in subgroups at this sample size. Seventh, ROI measurements were performed by a single reader and their reproducibility was not assessed, and the pooled “all parameters combined” measure weights four distinct imaging characteristics equally and should be read as a descriptive summary only, not as a validated composite endpoint.

Finally, radiation dose was reported but not systematically weighed against diagnostic benefit, which is an important factor for clinical implementation, particularly given the higher dose requirements of UHR scanning modes; we have outlined above how this should be addressed in the confirmatory study.

## 5. Conclusions

In this exploratory pilot study, slice thickness and ME level markedly influenced the quantitative and qualitative image quality of UHR PCD-CT in RC, with large effects within UHR mode. Three radiology residents rated most UHR reconstructions above the standard reference for overall quality and lesion conspicuity, and those at 60 and 70 keV also for MRF sharpness, despite lower SNR and higher noise, a comparison that is confounded by differences in contrast phase and acquisition parameters. Among the settings tested, 3 mm at 60 keV received the highest proportion of favorable ratings in the descriptive pooled summary and is one candidate working reconstruction for future validation; 2 mm at 70 keV, with a closely comparable proportion, remains a plausible alternative, and a thin-slice series (e.g., 1 mm) could be evaluated as a complement. These findings narrow down the reconstruction settings to be tested in a diagnostic study of UHR PCD-CT against MRI and histopathology; they do not establish diagnostic benefit, and they do not support the use of UHR PCD-CT as a substitute for MRI.

## Figures and Tables

**Table 1:** CT acquisition and reconstruction parameters.

Parameter	Portal Venous Phase	Pelvic (UHR) Phase
**Acquisition parameters**		
Absolute delay (s)	77	40
Collimation (*n* × mm)	144 × 0.4	120 × 0.2
Tube voltage (kVp)	120	120
Quality reference (mAs)	121	225
CARE keV IQ level	170	250
Rotation time (ms)	500	500
Pitch	0.80	0.80
Scan direction	cranio-caudal	cranio-caudal
Breathing instruction	inspiration	inspiration
**Reconstruction parameters**		
Slice thickness (mm)	1 (standard reference: 3)	0.6/variable
Slice increment (mm)	0.8	0.4/variable
Reconstruction kernel	Br40	Br48
Iterative reconstruction	QIR 3	QIR 3
Matrix size	512	768
Field of view (mm)	individual	individual
Monoenergetic level (keV)	60	variable

The standard reference reconstruction used for comparison was the 3 mm, 60 keV reconstruction of the portal venous phase; the sixteen study reconstructions (0.6, 1, 2 and 3 mm at 45, 50, 60 and 70 keV) were derived from the pelvic UHR phase. Rows in bold are subheadings. keV, kiloelectron volt; mAs, milliampere-seconds; QIR, quantum iterative reconstruction; UHR, ultra-high resolution.

**Table 2:** Patient demographics and tumor characteristics.

Variable	Value
**Patient demographics (*n* = 19)**	
Age (years)	61.7 ± 10.7
BMI (kg/m^2^)	24.9 (22.2–27.5)
Sex (male)	14 (73.7%)
**Tumor characteristics (*n* = 19)**	
*Histology*	
Rectal adenocarcinoma	17 (89.5%)
High-grade intraepithelial neoplasia	1 (5.3%)
Anal squamous cell carcinoma	1 (5.3%)
*Tumor location*	
Lower third (0–6 cm)	8 (42.1%)
Middle third (6–12 cm)	8 (42.1%)
Upper third (12–16 cm)	3 (15.8%)
**Rectal adenocarcinoma (*n* = 17)**	
*Grade*	
1	5 (29.4%)
2	8 (47.1%)
3	1 (5.9%)
Not assessable	3 (17.6%)
*AJCC stage*	
I	1 (5.9%)
IIA	2 (11.8%)
IIIB	11 (64.7%)
IVA	2 (11.8%)
IVB	1 (5.9%)
*T category*	
T1	1 (5.9%)
T3, not further specified	8 (47.1%)
T3a	3 (17.6%)
T3b	3 (17.6%)
T3c	1 (5.9%)
T3d	1 (5.9%)
Positive N category	14 (82.4%)
Positive M category	3 (17.6%)
*Tumor morphology (staging MRI)*	
Polypoid	4 (23.5%)
Semi-annular	10 (58.8%)
Annular	3 (17.6%)
*Mesorectal fascia*	
Involved (≤1 mm)	5 (29.4%)
of which tumor abutting or invading fascia	3 (17.6%)
Free (>1 mm)	12 (70.6%)

Values are mean ± standard deviation, median (interquartile range) or absolute number (percentage). Mesorectal fascia involvement was defined as a tumor-to-fascia distance of ≤1 mm according to the updated ESGAR consensus recommendations [5]. Rows in bold denote patient groups, and rows in italics denote characteristics within these groups. AJCC, American Joint Committee on Cancer; BMI, body mass index; MRI, magnetic resonance imaging.

**Table 3:** Radiation dose metrics.

Variable	Value
**Arterial phase (upper abdomen, QuantumPlus)**	
CTDIvol (mGy)	8.7 ± 3.0
DLP (mGy·cm)	234.7 ± 87.0
SSDE (mGy)	10.8 ± 2.4
Scan length (mm)	230 (223–250)
**Portal venous phase (chest/abdomen/pelvis, QuantumPlus)**	
CTDIvol (mGy)	8.4 ± 2.5
DLP (mGy·cm)	556.4 ± 167.9
SSDE (mGy)	10.3 ± 1.9
Scan length (mm)	622.4 (613.5–646.8)
**Ultra-high-resolution phase (pelvis)**	
CTDIvol (mGy)	18.3 ± 2.9
DLP (mGy·cm)	343.0 ± 144.9
SSDE (mGy)	22.7 ± 2.9
Scan length (mm)	167 (128–200)
**Total DLP, all series (mGy·cm)**	**1134.6 ± 364.6**

Values are mean ± standard deviation or median (interquartile range). Rows in bold denote the acquisition phases and the total DLP of all series. CTDIvol, volume computed tomography dose index; DLP, dose length product; SSDE, size-specific dose estimate.

**Table 4:** Quantitative parameters by slice thickness and monoenergetic level.

**Mode**	**ST (mm)**	**keV**	**SNR**	**CNR**	**Noise (HU)**
UHR	0.6	45	6.20 (1.59)	3.35 (1.63)	21.03 (3.31)
UHR	0.6	50	5.85 (1.44)	2.69 (1.42)	19.50 (3.12)
UHR	0.6	60	5.37 (1.29)	1.63 (1.17)	17.08 (2.80)
UHR	0.6	70	5.29 (1.29)	0.86 (1.04)	14.78 (2.51)
UHR	1.0	45	6.25 (1.57)	3.38 (1.65)	20.89 (3.13)
UHR	1.0	50	5.89 (1.43)	2.72 (1.45)	19.40 (2.94)
UHR	1.0	60	5.39 (1.24)	1.64 (1.17)	17.01 (2.62)
UHR	1.0	70	5.32 (1.25)	0.87 (1.06)	14.69 (2.44)
UHR	2.0	45	8.02 (2.01)	4.31 (2.15)	16.16 (2.31)
UHR	2.0	50	7.58 (1.81)	3.48 (1.88)	14.94 (2.17)
UHR	2.0	60	6.95 (1.57)	2.08 (1.53)	13.05 (1.92)
UHR	2.0	70	6.74 (1.65)	1.08 (1.38)	11.53 (1.98)
UHR	3.0	45	9.03 (2.31)	4.85 (2.43)	14.35 (2.10)
UHR	3.0	50	8.57 (2.08)	3.92 (2.13)	13.21 (1.95)
UHR	3.0	60	7.89 (1.85)	2.37 (1.75)	11.53 (1.77)
UHR	3.0	70	7.67 (1.92)	1.21 (1.58)	10.15 (1.81)
QuantumPlus	3.0	60	11.12 (2.82)	2.73 (2.12)	8.30 (1.34)

Values are mean (standard deviation). CNR, contrast-to-noise ratio; HU, Hounsfield units; keV, kiloelectron volt; SNR, signal-to-noise ratio; ST, slice thickness; UHR, ultra-high resolution.

**Table 5:** Mixed-effects model results for quantitative parameters.

Parameter	Factor	F (df)	*p* Value	η^2^p	Effect
CNR	Slice thickness	43.58 (3, 270)	<0.001	0.33	Main effect
	ME level	97.72 (3, 270)	<0.001	0.52	Main effect
	ST × ME level	5.11 (9, 270)	<0.001	0.15	Interaction
SNR	Slice thickness	181.13 (3, 270)	<0.001	0.67	Main effect
	ME level	17.31 (3, 270)	<0.001	0.16	Main effect
	ST × ME level	0.98 (9, 270)	0.455	0.03	Interaction not significant
Noise	Slice thickness	341.59 (3, 270)	<0.001	0.79	Main effect
	ME level	225.96 (3, 270)	<0.001	0.72	Main effect
	ST × ME level	6.53 (9, 270)	<0.001	0.18	Interaction

CNR, contrast-to-noise ratio; df, degrees of freedom; ME, monoenergetic; SNR, signal-to-noise ratio; ST, slice thickness; η^2^p, partial eta squared.

**Table 6:** Estimated marginal means and differences in quantitative parameters by slice thickness and monoenergetic level. (**a**) Estimated marginal means (95% confidence interval). (**b**) Differences between levels (95% confidence interval).

(**a**)				
**Factor**	**Level**	**SNR**	**CNR**	**Noise (HU)**
Slice thickness (mm), averaged over ME levels	0.6	5.68 (5.01–6.35)	2.13 (1.51–2.76)	18.10 (16.70–19.49)
	1	5.71 (5.06–6.37)	2.15 (1.52–2.79)	18.00 (16.67–19.32)
	2	7.32 (6.48–8.16)	2.74 (1.91–3.56)	13.92 (12.93–14.91)
	3	8.29 (7.32–9.26)	3.09 (2.15–4.03)	12.31 (11.41–13.21)
ME level (keV), averaged over slice thicknesses	45	7.38 (6.48–8.27)	3.97 (3.02–4.92)	18.11 (16.82–19.40)
	50	6.97 (6.16–7.78)	3.20 (2.38–4.03)	16.76 (15.55–17.97)
	60	6.40 (5.69–7.11)	1.93 (1.25–2.61)	14.67 (13.59–15.75)
	70	6.26 (5.53–6.99)	1.01 (0.40–1.61)	12.79 (11.74–13.83)
(**b**)				
**Factor**	**Contrast**	**ΔSNR**	**ΔCNR**	**ΔNoise (HU)**
Slice thickness (mm), averaged over ME levels	0.6 vs. 3 mm	−2.62 (−2.98 to −2.25)	−0.95 (−1.28 to −0.63)	5.79 (5.13–6.44)
	1 vs. 3 mm	−2.58 (−2.92 to −2.24)	−0.93 (−1.25 to −0.62)	5.68 (5.18–6.19)
	2 vs. 3 mm	−0.97 (−1.13 to −0.82)	−0.35 (−0.47 to −0.23)	1.61 (1.45–1.77)
ME level (keV), averaged over slice thicknesses	45 vs. 60 keV	0.98 (0.70–1.25)	2.04 (1.70–2.38)	3.44 (3.05–3.83)
	50 vs. 60 keV	0.57 (0.40–0.75)	1.27 (1.07–1.48)	2.09 (1.82–2.37)
	70 vs. 60 keV	−0.14 (−0.26 to −0.02)	−0.92 (−1.05 to −0.80)	−1.88 (−2.19 to −1.57)
Selected contrasts	1 vs. 0.6 mm	0.03 (−0.06 to 0.13)	0.02 (−0.03 to 0.07)	−0.10 (−0.42 to 0.22)
	3 mm/60 keV vs. 0.6 mm/70 keV	2.61 (2.27–2.94)	1.50 (1.14–1.87)	−3.25 (−3.74 to −2.75)

Values are means and mean differences averaged over the levels of the other factor (equal to the estimated marginal means of the linear mixed-effects models, as the design was complete and balanced), with 95% confidence intervals calculated from the 19 per-patient values (t distribution, 18 degrees of freedom), unadjusted for multiplicity. CNR, contrast-to-noise ratio; HU, Hounsfield units; ME, monoenergetic; SNR, signal-to-noise ratio.

**Table 7:** Effects of slice thickness and monoenergetic level on qualitative parameters.

Parameter	Factor	χ^2^ (df)	*p* Value	Effect
Overall image quality	ME level	213.67 (3)	<0.001	Main effect
	Slice thickness	65.93 (3)	<0.001	Main effect
	ME level × ST	10.09 (9)	0.344	Interaction not significant
Perceived noise	ME level	81.18 (3)	<0.001	Main effect
	Slice thickness	216.31 (3)	<0.001	Main effect
	ME level × ST	2.62 (9)	0.978	Interaction not significant
Mesorectal fascia sharpness	ME level	288.24 (3)	<0.001	Main effect
	Slice thickness	5.92 (3)	0.115	Not significant
	ME level × ST	3.36 (9)	0.948	Interaction not significant
Lesion conspicuity	ME level	71.30 (3)	<0.001	Main effect
	Slice thickness	3.20 (3)	0.361	Not significant
	ME level × ST	14.22 (9)	0.115	Interaction not significant

Effects were assessed by cumulative link mixed models with crossed random intercepts for patient and reader and tested by likelihood-ratio tests against the corresponding nested model; χ^2^ and degrees of freedom are given. Random-intercept standard deviations (patient; reader) were 1.13; 1.23 for overall image quality, 0.96; 0.60 for perceived noise, 0.98; 0.41 for mesorectal fascia sharpness and 1.32; 0.85 for lesion conspicuity. df, degrees of freedom; ME, monoenergetic; ST, slice thickness.

## Data Availability

The data presented in this study are available on request from the corresponding author due to privacy restrictions on patient data.

## Supplementary Materials

The following supporting information can be downloaded at https://www.mdpi.com/article/10.3390/diagnostics16213697/s1. Table S1: Likert scale definitions for qualitative image assessment; Table S2: Distribution of qualitative ratings relative to the standard reference; Table S3: Inter-reader agreement; Table S4: Sensitivity analysis after exclusion of the two patients whose final diagnosis was not rectal adenocarcinoma (*n* = 17); Table S5: Proportion of favorable ratings with 95% confidence intervals for each reconstruction; Figure S1: Patient accrual flowchart.
