# Supplementary material for: Ultra-High-Resolution Imaging of Rectal Cancer with Photon-Counting Detector Computed Tomography: An Exploratory Pilot Study on the Impact of Slice Thickness and Monoenergetic Reconstruction Level on Image Quality
Source: Diagnostics (Basel). 2026 Nov 9;16(21):3697. doi: 10.3390/diagnostics16213697 (PMC13762976; doi:10.3390/diagnostics16213697)
Supplement: Supplementary file 1 [file diagnostics-16-03697-s001.zip › diagnostics-4639105-supplementary.pdf]

diagnostics

MDPI

Supplementary Materials

Ultra-High-Resolution Imaging of Rectal Cancer with Photon-Counting Detector Computed Tomography: Impact of Slice Thickness and Monoenergetic Reconstruction Level on Image Quality—An Exploratory Pilot Study

Alexander Herold [1], Andreas Strassl [1,2], Daniela Beitzke [1], Philipp Stelzer [1], Christian Wassipaul [1], Theresa Dachs [1], Bence Nemeth [1], Florian Lindenlaub [1], Ahmed Ba-Ssalamah [1], Johannes Längle [3], Michael Bergmann [3], Michael Weber [1], Dietmar Tamandl [1,]* and Michael A. Arnoldner [1]

1 Department of Biomedical Imaging and Image-Guided Therapy, Medical University of Vienna, Waehringer Guertel 18-20, 1090 Vienna, Austria
2 Christian Doppler Laboratory for Machine Learning Driven Precision Medicine, Department of Biomedical Imaging and Image-Guided Therapy, Medical University of Vienna, Waehringer Guertel 18-20, 1090 Vienna, Austria
3 Division of Visceral Surgery, Department of General Surgery, Comprehensive Cancer Center Vienna, Medical University of Vienna, Waehringer Guertel 18-20, 1090 Vienna, Austria
* Correspondence: dietmar.tamandl@meduniwien.ac.at; Tel.: +43-1-40400-48000

Table S1. Likert scale definitions for qualitative image assessment.

Rating	Overall Image Quality	Perceived Noise	Mesorectal Fascia Sharpness	Lesion Conspicuity
+2	Markedly superior image quality	Markedly less noise	Markedly sharper fascia delineation	Markedly better lesion visibility
+1	Moderately superior image quality	Moderately less noise	Moderately sharper fascia delineation	Moderately better lesion visibility
0	Equivalent image quality	Equivalent noise level	Equivalent fascia delineation	Equivalent lesion visibility
−1	Moderately inferior image quality	Moderately more noise	Moderately less sharp fascia delineation	Moderately reduced lesion visibility
−2	Markedly inferior image quality	Markedly more noise	Markedly less sharp fascia delineation	Markedly reduced lesion visibility

All ratings were made relative to the standard reference reconstruction (QuantumPlus, 3.0 mm slice thickness, 60 keV).

Table S2. Distribution of qualitative ratings relative to the standard reference.

Parameter	ST (mm)	keV	−2 (%)	−1 (%)	0 (%)	+1 (%)	+2 (%)	n
Overall image quality	0.6	45	24.6	19.3	15.8	40.4	0.0	57
	0.6	50	12.3	29.8	12.3	45.6	0.0	57
	0.6	60	5.3	22.8	7.0	61.4	3.5	57
	0.6	70	0.0	8.8	12.3	73.7	5.3	57
	1	45	15.8	24.6	15.8	43.9	0.0	57
	1	50	10.5	28.1	19.3	42.1	0.0	57
	1	60	1.8	22.8	7.0	66.7	1.8	57
	1	70	0.0	12.3	14.0	68.4	5.3	57
	2	45	3.5	28.1	22.8	45.6	0.0	57
	2	50	3.5	22.8	19.3	54.4	0.0	57
	2	60	0.0	10.5	12.3	73.7	3.5	57

Parameter	ST (mm)	keV	−2 (%)	−1 (%)	0 (%)	+1 (%)	+2 (%)	n
	2	70	0.0	3.5	5.3	78.9	12.3	57
	3	45	1.8	26.3	19.3	52.6	0.0	57
	3	50	5.3	14.0	19.3	59.6	1.8	57
	3	60	0.0	3.5	10.5	68.4	17.5	57
	3	70	0.0	3.5	8.8	73.7	14.0	57
Perceived noise	0.6	45	57.9	33.3	5.3	3.5	0.0	57
	0.6	50	61.4	29.8	3.5	5.3	0.0	57
	0.6	60	31.6	61.4	3.5	3.5	0.0	57
	0.6	70	22.8	64.9	7.0	5.3	0.0	57
	1	45	59.6	31.6	7.0	1.8	0.0	57
	1	50	54.4	33.3	10.5	1.8	0.0	57
	1	60	38.6	54.4	5.3	1.8	0.0	57
	1	70	22.8	63.2	10.5	3.5	0.0	57
	2	45	29.8	43.9	22.8	3.5	0.0	57
	2	50	24.6	57.9	10.5	7.0	0.0	57
	2	60	8.8	63.2	22.8	5.3	0.0	57
	2	70	7.0	50.9	36.8	5.3	0.0	57
	3	45	19.3	47.4	31.6	1.8	0.0	57
	3	50	15.8	45.6	33.3	5.3	0.0	57
	3	60	8.8	42.1	35.1	14.0	0.0	57
	3	70	1.8	36.8	50.9	10.5	0.0	57
Mesorectal fascia sharpness	0.6	45	1.8	17.5	43.9	33.3	3.5	57
	0.6	50	1.8	12.3	40.4	42.1	3.5	57
	0.6	60	0.0	3.5	10.5	77.2	8.8	57
	0.6	70	0.0	0.0	10.5	77.2	12.3	57
	1	45	7.0	22.8	38.6	29.8	1.8	57
	1	50	3.5	19.3	33.3	42.1	1.8	57
	1	60	0.0	8.8	14.0	71.9	5.3	57
	1	70	0.0	3.5	14.0	68.4	14.0	57
	2	45	1.8	14.0	45.6	38.6	0.0	57
	2	50	5.3	3.5	49.1	42.1	0.0	57
	2	60	0.0	0.0	24.6	64.9	10.5	57
	2	70	0.0	0.0	12.3	73.7	14.0	57
	3	45	1.8	8.8	73.7	15.8	0.0	57
	3	50	1.8	8.8	40.4	49.1	0.0	57
	3	60	0.0	1.8	17.5	71.9	8.8	57
	3	70	0.0	1.8	14.0	70.2	14.0	57
Lesion conspicuity	0.6	45	0.0	1.8	19.3	49.1	29.8	57
	0.6	50	0.0	3.5	12.3	57.9	26.3	57
	0.6	60	0.0	1.8	12.3	78.9	7.0	57
	0.6	70	0.0	1.8	28.1	63.2	7.0	57
	1	45	1.8	1.8	12.3	57.9	26.3	57
	1	50	1.8	0.0	17.5	59.6	21.1	57
	1	60	0.0	1.8	19.3	71.9	7.0	57
	1	70	0.0	3.5	24.6	68.4	3.5	57
	2	45	0.0	1.8	12.3	54.4	31.6	57
	2	50	0.0	1.8	17.5	56.1	24.6	57
	2	60	0.0	0.0	12.3	82.5	5.3	57
	2	70	0.0	0.0	19.3	80.7	0.0	57
	3	45	0.0	1.8	14.0	57.9	26.3	57
	3	50	0.0	3.5	10.5	61.4	24.6	57
	3	60	0.0	0.0	8.8	66.7	24.6	57
	3	70	0.0	0.0	35.1	64.9	0.0	57
All parameters combined	0.6	45	21.1	18.0	21.1	31.6	8.3	228
	0.6	50	18.9	18.9	17.1	37.7	7.5	228
	0.6	60	9.2	22.4	8.3	55.3	4.8	228
	0.6	70	5.7	18.9	14.5	54.8	6.1	228
	1	45	21.1	20.2	18.4	33.3	7.0	228
	1	50	17.5	20.2	20.2	36.4	5.7	228
	1	60	10.1	21.9	11.4	53.1	3.5	228
	1	70	5.7	20.6	15.8	52.2	5.7	228
	2	45	8.8	21.9	25.9	35.5	7.9	228

Parameter	ST (mm)	keV	−2 (%)	−1 (%)	0 (%)	+1 (%)	+2 (%)	n
	2	50	8.3	21.5	24.1	39.9	6.1	228
	2	60	2.2	18.4	18.0	56.6	4.8	228
	2	70	1.8	13.6	18.4	59.6	6.6	228
	3	45	5.7	21.1	34.6	32.0	6.6	228
	3	50	5.7	18.0	25.9	43.9	6.6	228
	3	60	2.2	11.8	18.0	55.3	12.7	228
	3	70	0.4	10.5	27.2	54.8	7.0	228

Values are percentages of ratings pooled across three readers and 19 patients (n = 57 ratings per parameter and reconstruction; n = 228 for all parameters combined); rows sum to 100%. Ratings were made on a five-point scale relative to the standard reference: −2, markedly inferior; −1, moderately inferior; 0, equivalent; +1, moderately superior; +2, markedly superior. keV, kiloelectron volt; ST, slice thickness.

Table S3. Inter-reader agreement.

Parameter	Gwet's AC2	95% CI
Overall image quality	0.716	0.677–0.754
Perceived noise	0.640	0.594–0.686
Mesorectal fascia sharpness	0.828	0.803–0.853
Lesion conspicuity	0.883	0.866–0.899

Values are Gwet's agreement coefficient version 2 with ordinal weights and 95% confidence intervals. AC2, Gwet's agreement coefficient version 2; CI, confidence interval.

Table S4. Sensitivity analysis after exclusion of the two patients whose final diagnosis was not rectal adenocarcinoma (n = 17).

Parameter	Factor	F (df)	p Value	η^2p
CNR	Slice thickness	47.26 (3, 240)	<0.001	0.37
	ME level	107.35 (3, 240)	<0.001	0.57
	ST × ME level	5.71 (9, 240)	<0.001	0.18
SNR	Slice thickness	171.43 (3, 240)	<0.001	0.68
	ME level	15.46 (3, 240)	<0.001	0.16
	ST × ME level	0.88 (9, 240)	0.539	0.03
Noise	Slice thickness	310.13 (3, 240)	<0.001	0.79
	ME level	208.88 (3, 240)	<0.001	0.72
	ST × ME level	6.14 (9, 240)	<0.001	0.19

(a) Mixed-effects model results for the quantitative parameters. CNR, contrast-to-noise ratio; df, degrees of freedom; ME, monoenergetic; SNR, signal-to-noise ratio; ST, slice thickness; η^2p, partial eta squared.

ST (mm)	keV	All patients (n = 19)	Adenocarcinoma only (n = 17)
0.6	45	39.9 (32.5–47.4)	42.2 (35.3–49.5)
0.6	50	45.2 (37.7–52.2)	46.6 (41.2–52.0)
0.6	60	60.1 (52.6–65.8)	62.3 (57.8–66.2)
0.6	70	61.0 (56.1–65.8)	61.3 (56.4–65.7)
1	45	40.4 (34.6–45.2)	42.6 (39.2–46.6)
1	50	42.1 (36.0–47.4)	44.6 (40.2–49.0)
1	60	56.6 (46.9–65.4)	59.3 (51.5–66.2)
1	70	57.9 (50.4–64.9)	57.8 (50.0–65.2)
2	45	43.4 (36.4–50.4)	44.6 (37.3–51.5)
2	50	46.1 (36.8–54.4)	48.0 (39.7–55.9)
2	60	61.4 (51.8–68.9)	65.7 (59.8–70.6)
2	70	66.2 (60.1–71.5)	66.7 (59.3–72.1)
3	45	38.6 (32.5–44.7)	39.2 (33.8–45.1)

ST (mm)	keV	All patients (n = 19)	Adenocarcinoma only (n = 17)
3	50	50.4 (40.4–59.6)	51.0 (42.6–59.3)
3	60	68.0 (58.8–75.9)	69.6 (62.7–75.5)
3	70	61.8 (53.9–69.3)	63.7 (56.9–70.6)

(b) Pooled proportion of favorable ratings (all parameters combined, %, with 95% cluster-bootstrap confidence interval) for each reconstruction in the full cohort and in the adenocarcinoma-only cohort. keV, kiloelectron volt; ST, slice thickness.

Table S5. Proportion of favorable ratings with 95% confidence intervals for each reconstruction.

Parameter	ST (mm)	keV	n	Favorable (%)	95% CI
Overall image quality	0.6	45	57	40.4	31.6–49.1
	0.6	50	57	45.6	33.3–56.1
	0.6	60	57	64.9	54.4–75.4
	0.6	70	57	78.9	70.2–87.7
	1	45	57	43.9	31.6–54.4
	1	50	57	42.1	31.6–52.6
	1	60	57	68.4	56.1–80.7
	1	70	57	73.7	57.9–87.7
	2	45	57	45.6	31.6–57.9
	2	50	57	54.4	38.6–70.2
	2	60	57	77.2	61.4–91.2
	2	70	57	91.2	82.5–98.2
	3	45	57	52.6	38.6–68.4
	3	50	57	61.4	45.6–75.4
	3	60	57	86.0	73.7–96.5
	3	70	57	87.7	78.9–96.5
	All	45	228	45.6	36.4–55.3
	All	50	228	50.9	40.8–60.5
	All	60	228	74.1	63.6–82.9
	All	70	228	82.9	75.4–89.0
Perceived noise	0.6	45	57	3.5	0.0–8.8
	0.6	50	57	5.3	0.0–10.5
	0.6	60	57	3.5	0.0–8.8
	0.6	70	57	5.3	0.0–10.5
	1	45	57	1.8	0.0–5.3
	1	50	57	1.8	0.0–5.3
	1	60	57	1.8	0.0–5.3
	1	70	57	3.5	0.0–8.8
	2	45	57	3.5	0.0–8.8
	2	50	57	7.0	1.8–14.0
	2	60	57	5.3	0.0–14.0
	2	70	57	5.3	0.0–10.5
	3	45	57	1.8	0.0–5.3
	3	50	57	5.3	0.0–14.0
	3	60	57	14.0	5.3–22.8
	3	70	57	10.5	3.5–17.5
	All	45	228	2.6	0.9–4.8
	All	50	228	4.8	2.2–8.3
	All	60	228	6.1	2.6–10.1
	All	70	228	6.1	2.6–10.1
Mesorectal fascia sharpness	0.6	45	57	36.8	26.3–47.4
	0.6	50	57	45.6	31.6–59.6
	0.6	60	57	86.0	75.4–94.7
	0.6	70	57	89.5	82.5–96.5
	1	45	57	31.6	24.6–38.6
	1	50	57	43.9	31.6–56.1
	1	60	57	77.2	61.4–89.5
	1	70	57	82.5	71.9–91.2
	2	45	57	38.6	24.6–54.4
	2	50	57	42.1	26.3–57.9

Parameter	ST (mm)	keV	n	Favorable (%)	95% CI
	2	60	57	75.4	63.2–86.0
	2	70	57	87.7	77.2–96.5
	3	45	57	15.8	7.0–24.6
	3	50	57	49.1	33.3–64.9
	3	60	57	80.7	68.4–91.2
	3	70	57	84.2	75.4–93.0
	All	45	228	30.7	22.4–39.0
	All	50	228	45.2	33.8–57.0
	All	60	228	79.8	69.7–87.3
	All	70	228	86.0	80.3–91.2
Lesion conspicuity	0.6	45	57	78.9	66.7–89.5
	0.6	50	57	84.2	70.2–94.7
	0.6	60	57	86.0	73.7–96.5
	0.6	70	57	70.2	61.4–79.0
	1	45	57	84.2	71.9–94.7
	1	50	57	80.7	68.4–91.2
	1	60	57	78.9	64.9–91.2
	1	70	57	71.9	57.9–86.0
	2	45	57	86.0	75.4–94.7
	2	50	57	80.7	66.7–91.2
	2	60	57	87.7	75.4–96.5
	2	70	57	80.7	70.2–89.5
	3	45	57	84.2	75.4–93.0
	3	50	57	86.0	71.9–96.5
	3	60	57	91.2	78.9–100.0
	3	70	57	64.9	49.1–78.9
	All	45	228	83.3	74.1–90.8
	All	50	228	82.9	70.6–92.1
	All	60	228	86.0	74.1–94.3
	All	70	228	71.9	61.8–81.1
All parameters combined	0.6	45	228	39.9	32.5–47.4
	0.6	50	228	45.2	37.7–52.2
	0.6	60	228	60.1	52.6–65.8
	0.6	70	228	61.0	56.1–65.8
	1	45	228	40.4	34.6–45.2
	1	50	228	42.1	36.0–47.4
	1	60	228	56.6	46.9–65.4
	1	70	228	57.9	50.4–64.9
	2	45	228	43.4	36.4–50.4
	2	50	228	46.1	36.8–54.4
	2	60	228	61.4	51.8–68.9
	2	70	228	66.2	60.1–71.5
	3	45	228	38.6	32.5–44.7
	3	50	228	50.4	40.4–59.6
	3	60	228	68.0	58.8–75.9
	3	70	228	61.8	53.9–69.3
	All	45	912	40.6	35.0–45.8
	All	50	912	45.9	38.7–52.3
	All	60	912	61.5	53.8–67.7
	All	70	912	61.7	56.5–66.0

Favorable ratings are ratings of +1 or +2 relative to the standard reference, pooled across three readers. The 95% confidence intervals were obtained by a cluster bootstrap that resampled patients together with all their ratings (2000 resamples, percentile method). Rows labeled "All" give the proportions pooled over the four slice thicknesses (n = 228 ratings; n = 912 for all parameters combined), as quoted in the Results. "All parameters combined" pools the four parameters with equal weighting as a descriptive summary; it is not a validated composite endpoint. keV, kiloelectron volt; ST, slice thickness.

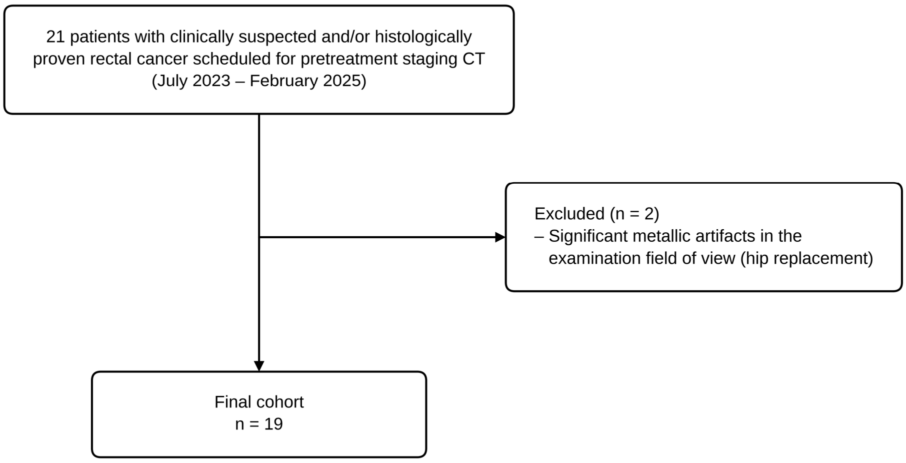

Figure S1. Patient accrual flowchart. CT, computed tomography.

https://doi.org/10.3390/diagnostics16213697
